# Hydropower reservoirs on the upper Mekong River modify nutrient bioavailability downstream

**DOI:** 10.1093/nsr/nwaa026

**Published:** 2020-02-17

**Authors:** Qiuwen Chen, Wenqing Shi, Jef Huisman, Stephen C Maberly, Jianyun Zhang, Juhua Yu, Yuchen Chen, Daniele Tonina, Qitao Yi

**Affiliations:** State Key Laboratory of Hydrology-Water Resources & Hydraulic Engineering, Nanjing Hydraulic Research Institute, Nanjing 210029, China; Center for Eco-Environment Research, Nanjing Hydraulic Research Institute, Nanjing 210098, China; State Key Laboratory of Hydrology-Water Resources & Hydraulic Engineering, Nanjing Hydraulic Research Institute, Nanjing 210029, China; Center for Eco-Environment Research, Nanjing Hydraulic Research Institute, Nanjing 210098, China; Department of Freshwater and Marine Ecology, Institute for Biodiversity and Ecosystem Dynamics, University of Amsterdam, Amsterdam 1090 GE, The Netherlands; Lake Ecosystems Group, UK Centre for Ecology & Hydrology, Lancaster LA1 4AP, UK; Department of Eco-Environment Conservation, Yangtze Institute for Conservation and Development, Nanjing 210029, China; Center for Eco-Environment Research, Nanjing Hydraulic Research Institute, Nanjing 210098, China; Center for Eco-Environment Research, Nanjing Hydraulic Research Institute, Nanjing 210098, China; Center for Ecohydraulics Research, University of Idaho, Boise, ID 83702, USA; Center for Eco-Environment Research, Nanjing Hydraulic Research Institute, Nanjing 210098, China

**Keywords:** Mekong River, cascade reservoirs, downstream nutrient bioavailability, nitrogen, phosphorus

## Abstract

Hydropower development is the key strategy in many developing countries for energy supply, climate-change mitigation and economic development. However, it is commonly assumed that river dams retain nutrients and therefore reduce downstream primary productivity and fishery catches, compromising food security and causing trans-boundary disputes. Contrary to expectation, here we found that a cascade of reservoirs along the upper Mekong River increased downstream bioavailability of nitrogen and phosphorus. The dams caused phytoplankton density to increase with hydraulic residence time and stratification of the stagnant reservoirs caused hypoxia at depth. This allowed the release of bioavailable phosphorus from the sediment and an increase in dissolved inorganic nitrogen as well as a shift in nitrogen species from nitrate to ammonium, which were transported downstream by the discharge of water from the base of the dam. Our findings provide a new perspective on the environmental impacts of river dams on nutrient cycling and ecosystem functioning, with potential implications for sustainable development of hydropower worldwide.

## INTRODUCTION

Hydropower is considered a major source of clean energy in many developing regions such as on the rivers Mekong, Congo and Amazon [1]. Many rivers have been intensively dammed for hydropower production, as well as flood management, water supply and navigation. More than 70 000 large dams have been built worldwide since the Industrial Revolution [2] and this number will continue to increase in the foreseeable future [3]. Dams convert rivers into lentic reservoirs and greatly modify the hydrological regimes by decreasing flow velocity, increasing hydraulic residence time and trapping sediments [4,5]. It is therefore commonly argued that reservoirs sequester nutrients [2,6], significantly reducing primary productivity, fishery catches and food security downstream [1,7,8]. The impacts of river damming on nutrient retention and ecosystem production have become a serious environmental issue in rivers and often cause tensions between nations along trans-boundary rivers, including the Mekong River [3,9,10], which seriously constrains the sustainable development of hydropower.

The Mekong River (named the Lancang River in China) originates in the Tibetan Plateau at an altitude of about 5200 m and flows 4909 km southeast to the South China Sea, through six developing countries: China, Myanmar, Laos, Thailand, Cambodia and Vietnam [9]. It supports the largest freshwater fishery in the world, providing a primary source of protein to over 55 million people [11]. At present, 21 hydropower reservoirs have been built, or are under construction or are being planned in the mainstream of the upper Mekong River [12]. By 2018, the installed capacity in the upper Mekong was 2.0 × 10^6^ kW and the annual production was 0.9 × 10^12^ kW h (see Fig. 1 in the Supplementary data). Meanwhile, hydropower dams on the upper Mekong River have been blamed for decreased fishery catches downstream, which has been attributed to nutrient retention and consequently reduced phytoplankton productivity [7–9]. However, these negative views are often based either on simulation results from models [2,6] or on presumptions based on the observed fishery decline [3,8,13], rather than on direct scientific evidence. The disputes have affected public opinion and harmed the sustainable development of Mekong-basin countries.

This study investigated changes in nutrient availability and phytoplankton-community structure in the cascade hydropower reservoirs along the upper Mekong River based on extensive and long-term monitoring programs. The major contributions are that we: (i) showed that hydropower dams stimulate phytoplankton production and increase the bioavailability of nutrients downstream and (ii) revealed the underlying mechanisms associated with nutrient transformations after river damming.

## RESULTS

### Long-term changes in nutrients at the China–Myanmar border

**Figure 1.**
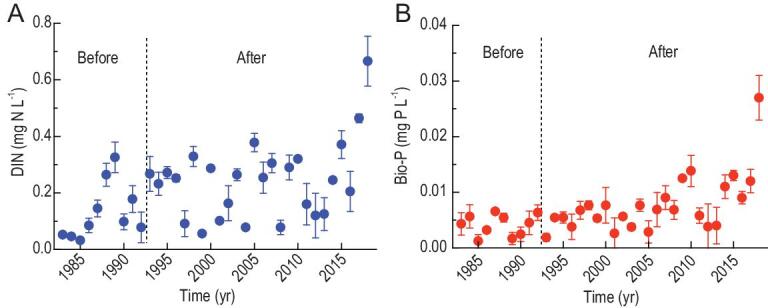
Long-term changes in nutrient concentration at the China–Myanmar border monitoring station. (A) DIN. (B) Bio-P. DIN, dissolved inorganic nitrogen; Bio-P, bioavailable phosphorus. The figure shows annual means of discharge-weighted nutrient concentrations based on three values per year in the 1980s (high flow, normal flow, low flow), four values in the 1990s (seasonally) and monthly values since 2000 (error bars show standard deviation). The data were collected from the Chinese Hydrological Year Book and the Hydrology and Water Resource Bureau of Yunnan Province, China. The trans-boundary monitoring station was located at Jinghong Station (1983–2007) and Guanlei Station (since 2008) (Fig. 6). The dashed vertical line shows the date of construction of the first reservoir.

The long-term observation data (Fig. 1) at the trans-boundary monitoring station showed that there has been no decline in bioavailable nitrogen (N) and phosphorus (P) downstream of six dams in the upper Mekong River. Before dam construction (1993), the concentrations of dissolved inorganic nitrogen (DIN) and bioavailable P was relatively stable at 0.03−0.34 and 0.001−0.007 mg L^−1^, respectively. After dam construction, there was a slight increase in both DIN and bioavailable P, which reached 0.67 and 0.027 mg L^−1^ in 2018, respectively.

### Changes in the concentration and composition of DIN down the reservoir cascade

**Figure 2.**
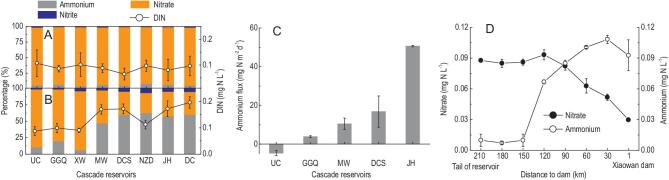
Concentration and composition of dissolved inorganic nitrogen in the water and ammonium flux across the sediment–water interface along the upper Mekong River. (A) DIN concentration and composition in the surface water before cascade hydropower development. (B) DIN concentration and composition in the water samples from the discharge of power station outlet right after a dam. (C) Ammonium flux across the sediment–water interface. (D) Changes in nitrate and ammonium concentrations in surface water from the tail to the front of the Xiaowan Reservoir. GGQ (Gongguoqiao), XW (Xiaowan), MW (Manwan), DCS (Dachaoshan), NZD (Nuozhadu) and JH (Jinghong) are the cascade reservoirs along the flow direction in the upper Mekong River. UC, upstream channel; DC, downstream channel; DIN, dissolved inorganic nitrogen. Data after hydropower development were collected in September 2016 and 2017, while data before hydropower development (August 1992) were collected from the Chinese Hydrological Year Book and the Hydrology and Water Resource Bureau of Yunnan Province, China. Error bars indicate standard deviation (*n* = 3).

Before hydropower development, the concentration of DIN was relatively constant at 0.06−0.11 mg N L^−1^ at all stations along the upper Mekong River and nitrate was the dominant DIN form (92.7%) at all sites down the reservoir cascade (Fig. 2A). After hydropower development, however, the concentration of DIN generally increased from 0.09 mg N L^−1^ in the upstream channel to 0.20 mg N L^−1^ in the downstream channel (Fig. 2B). The dominant form of inorganic N shifted from nitrate upstream to ammonium downstream: ammonium accounted for 10.8% of DIN in the upstream channel, but 60.0% in the downstream channel (Fig. 2B). The ammonium flux across the sediment–water interface was slightly negative in the upstream channel, indicating sediment uptake, but was positive (indicating sediment release) in the Gongguoqiao Reservoir and then increased further in the downstream reservoirs to a maximum of 50.6 mg N m^−2^ d^−1^ in the Jinghong Reservoir (Fig. 2C). In the Xiaowan Reservoir, a remarkable decrease in nitrate concentration and increase in ammonium concentration was observed along the flow direction. From the tail to the front of the reservoir, the nitrate concentration decreased from 0.09 to 0.03 mg N L^−1^, while the ammonium concentration increased from 0.01 to 0.09 mg N L^−1^ (Fig. 2D).

### Changes in the concentration and composition of bioavailable P down the reservoir cascade

**Figure 3.**
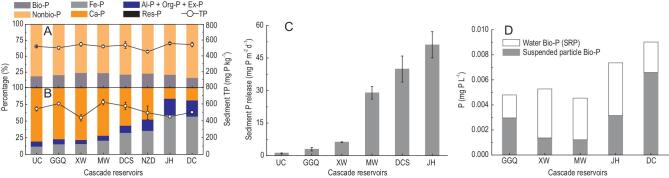
P fractions in sediments, P fluxes from sediments and bioavailable P concentration in the discharge from cascade reservoirs. (A) Bioavailable P ratio in sediments before cascade hydropower development. (B) P fractions in sediments after cascade hydropower development. Bio-P, bioavailable phosphorus; SRP, soluble reactive phosphorus. Bio-P includes iron-bound P (Fe–P), aluminum-bound P (Al–P), organic-bound P (Org–P) and exchangeable P (Ex-P), whereas non-bioavailable phosphorus includes calcium-bound P (Ca–P) and residual P (Res-P). (C) P fluxes from sediments. (D) Bioavailable P concentration, which is the sum of SRP and Bio-P in suspended particles per unit volume, in the water samples from the discharge of the power station outlet right after a dam. GGQ (Gongguoqiao), XW (Xiaowan), MW (Manwan), DCS (Dachaoshan), NZD (Nuozhadu) and JH (Jinghong) are cascade reservoirs along the flow direction in the upper Mekong River. UC, upstream channel; DC, downstream channel. In Fig. 3D, Bio-P data after hydropower development were collected in September 2016 and 2017, while bioavailable P before hydropower development were collected from the Chinese Hydrological Year Book and the Hydrology and Water Resource Bureau of Yunnan Province, China. Error bars indicate standard deviation (*n* = 3).

Total P in bed sediments did not differ significantly among sites before and after cascade hydropower development (one-way analysis of variance (ANOVA), *F*_6,12_ = 3.56, *P* > 0.05), which varied between 436 and 621 mg P kg^−1^ dry sediment (Fig. 3A and B). Before cascade hydropower development, the bioavailable P in the sediment was on average 21.2% of total P and similar at all stations along the upper Mekong River (Fig. 3A). However, the bioavailable P in the sediment showed a remarkable increase along the flow direction after cascade hydropower development and was larger than non-bioavailable P at the downstream sites. From the upstream to downstream channels, the bioavailable P as a percentage of total P increased from 22.0% to 83.7% (Fig. 3B), with a remarkable shift from Ca–P in the upstream channel to Fe–P in the downstream reservoirs. P fluxes across the sediment–water interface indicated that there was a large increase in sediment P release, from 1.1 mg P m^−2^ d^−1^ in the upstream channel to 51.1 mg P m^−2^ d^−1^ at the Jinghong Reservoir (Fig. 3C). In the tail water from hydropower stations, the bioavailable P concentration showed a general increase from 0.005 mg P L^−1^ in the Gongguoqiao Reservoir to 0.009 mg P L^−1^ in the downstream channel (Fig. 3D).

### Spatial patterns of phytoplankton abundance and composition

**Figure 4.**
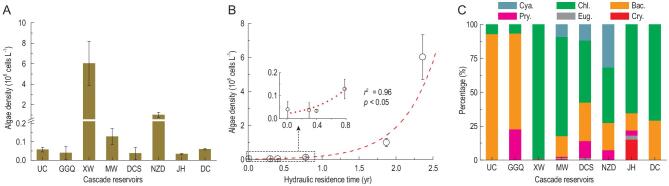
Phytoplankton communities in cascade reservoirs along the upper Mekong River. (A) Phytoplankton abundance. (B) Relationship between phytoplankton abundance and hydraulic residence time. (C) Phytoplankton composition. GGQ (Gongguoqiao), XW (Xiaowan), MW (Manwan), DCS (Dachaoshan), NZD (Nuozhadu) and JH (Jinghong) are the cascade reservoirs along the flow direction in the upper Mekong River. UC, upstream channel; DC, downstream channel; Cya., *Cyanobacteria*; Chl., *Chlorophyta*; Bac., *Bacillariophyta*; Pyr., *Pyrrophyta*; Eug., *Euglenophyta*; Cry., *Cryptophyta*. Data were collected in September 2016 and 2017. Error bars indicate standard deviation (*n* = 3).

Phytoplankton abundance was much higher in some of the reservoirs than in the upstream channel, particularly in large reservoirs such as Xiaowan and Nuozhadu (Fig. 4A). Regression analysis showed that there was a positive exponential relationship (*r*^2^ = 0.96, *n* = 6, *P* < 0.05) between phytoplankton abundance and reservoir hydraulic residence time (Fig. 4B). Phytoplankton abundance reached a maximum of 6.02 × 10^6^ cell L^−1^ in the Xiaowan Reservoir, which was about 100 times greater than that in the upstream channel (Fig. 4A). The dominant phytoplankton functional group shifted from diatoms (*Bacillariophyta*) to green algae (*Chlorophyta*) along the flow direction (Fig. 4C). From the upstream channel and Gongguoqiao Reservoir to the subsequent reservoirs and downstream channel, the percentage of diatoms decreased from 70.7%−93.1% to 0.3%−29.2%, whereas the percentage of green algae increased from 6.5%−6.9% to 41.0%−99.1%. In addition, *Cyanobacteria* genera, including *Microcystis*, *Dolichospermum* (formerly known as *Anabaena*), *Merismopedia* and *Spirulina*, made up a considerable fraction of the phytoplankton community in three of the downstream reservoirs (Fig. 4C).

## DISCUSSION

### Alteration of hydrological and mixing regime

Dams convert rivers into a series of lentic reservoirs with a subsequent decrease in flow velocity and mixing and an increase in hydraulic residence time [14]. In the upper Mekong River, hydraulic residence times of the Gongguoqiao, Xiaowan, Manwan, Dachaoshan, Nuozhadu and Jinghong reservoirs produce a total residence time of 5.72 years (see Table 1 in the Supplementary data), which greatly exceeds the residence time of <0.07 years before the dams were built. The longer residence time allows phytoplankton biomass to increase and the decreased mixing and greater depth increases settlement of suspended particles in the reservoirs; both led to higher organic-matter accumulation in reservoirs. The content of organic carbon in the reservoir sediment was about 4.6 times higher than that in the upstream channel (see Fig. 2 in the Supplementary data). Decomposition of settled organic carbon by microorganisms consumes oxygen in the bottom water and the weak vertical mixing in the stratified waters of the reservoirs restricts the supply of oxygen from the surface to the bottom water [15]. As a result, oxygen concentrations in the hypolimnion often become low enough for hypoxia to occur (see Fig. 3 in the Supplementary data) [4], which alters the biogeochemical cycling of nutrients and their exchange between sediment and water, and their transport downstream.

### Modification of nitrogen forms and increase of P bioavailability

In aquatic ecosystems, DIN is usually the most bioavailable N form and is regulated by various

transformations (e.g. phytoplankton assimilation, ammonification, nitrification, denitrification) [16–19]. In unregulated river systems with natural flow regimes, rivers tend to act as pipes for N transport from terrestrial to coastal systems with limited N transformation because of the short residence time [20]. The DIN composition in the upper Mekong River was relatively stable before cascade hydropower development (Fig. 2A). After river damming, the increased residence time altered N transformations and the DIN composition shifted towards ammonium along the direction of flow (Fig. 2B). In particular, nitrate taken up by bacteria and phytoplankton in the surface waters of stagnant reservoirs may have been transported to depth as particulate organic nitrogen. In the thermocline and deep bottom waters of stagnant reservoirs, the occurrence of hypoxia limits aerobic nitrification [21], leading to ammonium accumulation (Fig. 2C). Accordingly, the nitrate concentration in the Xiaowan surface water showed a large decrease from the tail to the front of the reservoir, while the ammonium concentration increased (Fig. 2D). Down the reservoir cascade, nitrate consumption in the surface water and ammonium accumulation in the deeper water layers contributed to the shift of the dominant DIN form from nitrate to ammonium after passing through the cascade reservoirs (Fig. 2B).

P is another essential nutrient for the primary productivity of aquatic ecosystems [22]. Bioavailable P (exchangeable P, iron-bound P and organic-bound P) in sediments is a major contributor to dissolved P in the water column [23], as it can be released from the sediment by a range of processes, including low dissolved oxygen concentration linked to sediment redox potential [24]. Along the upper Mekong River, there was a large shift in sediment P fractions from calcium-bound P in the upstream channel to iron-bound P in the downstream reservoirs. The occurrence of anoxia in reservoir sediments would have led to reductive dissolution of iron-bound P (Fig. 3C).

Dams are often assumed to retain sediment-bound nutrients and prevent them from moving downstream. For instance, based on estimates of the amount of sediment trapped in the reservoir, it has been calculated that the Xayaburi reservoir in the lower Mekong would retain about 33% of total nitrogen and 40% of total phosphorus [25,26]. However, our data show that total nitrogen and total phosphorus do not decline substantially along the cascade reservoirs in the upper Mekong (see Fig. 4 in the Supplementary data). Besides nutrient input from the drainage area along the river, there are two other major reasons for this discrepancy between previous calculations and our observations. First, the previous calculations [25,26] focused on nitrogen and phosphorus associated with suspended sediment particles. The sediments in the lower Mekong region are likely to have higher organic carbon and nutrient contents, which are trapped by the Xayaburi hydropower dam, whereas the upper Mekong region has a narrow and rocky catchment with less soil erosion and hence a low organic fraction of the sediments (see Fig. 2 in the Supplementary data) that contain less bioavailable nutrients. Second, and more importantly, the previous calculations [25,26] ignored the biogeochemical transformations of N and P in the oxygen-depleted sediments of the reservoirs, which resulted in the release of ammonium and bioavailable P from the trapped sediments into the water and the subsequent efflux of these nutrients at the base of the reservoir.

### Stimulation of phytoplankton production and shifts of community composition

The longer residence time and enhanced bioavailability of nutrients after the building of the dams stimulate phytoplankton growth. The positive exponential relationship between phytoplankton abundance and reservoir hydraulic residence time shown here demonstrates how large reservoirs can increase primary production in dammed rivers.

Our data showed a major shift in phytoplankton-species composition along the cascade of reservoirs, from predominantly diatoms in the upstream channel and upstream reservoir to predominantly green algae in the downstream reservoirs (Fig. 4C). This shift in phytoplankton-community structure is most likely associated with the relatively low availability of silicon, an essential nutrient for diatoms, in the Xiaowan Reservoir (see Fig. 5 in the Supplementary data), the gradually increased water temperature downstream (see Fig. 6A in the Supplementary data) as well as with increased sinking losses of the denser diatoms due to low mixing rates in the stagnant reservoirs. Indeed, diatoms are known to have a general preference for the relatively cold and turbulent lotic waters of rivers and streams, whereas many green algae grow well in lentic habitats [27]. The dominant form of DIN shifted from nitrate to ammonium, which is more bioavailable than nitrate. The increase in water temperature and hydraulic residence time was possibly also the mechanism that caused the appearance of cyanobacteria in the phytoplankton communities of downstream reservoirs [28,29]. Several studies have highlighted the effects of dams on silicon retention and the resulting consequences for phytoplankton communities in rivers [30]. It is estimated that 2.6% of the dissolved silicon (163 Gmol yr^−1^) loadings to the global river network are retained in reservoirs [30]. In this study, dissolved silicon was indeed retained by the most upstream reservoir Gongguoqiao (see Fig. 5 in the Supplementary data), which had high diatom abundance (Fig. 4). The subsequent Xiaowan Reservoir had a relatively low dissolved silicon concentration and diatoms were completely replaced by green algae (Fig. 4). In reservoirs farther downstream, the dissolved silicon concentration gradually recovered, probably due to inputs from tributaries draining sub-catchments with silicon-rich rocks in the upper Mekong River, which led to a mixture of diatoms, green algae and other phytoplankton. The increased nutrient bioavailability, water temperature and hydraulic residence time in combination with reduced turbulent mixing are likely to favor green algae and cyanobacteria over diatoms [29,31,32], consistently with the observed changes in phytoplankton-community structure along the downstream gradient.

### Implications for sustainable regional development

There has been much debate about the potential effects of the cascade dams in the upper Mekong River on nutrient retention and hence fishery collapse in the lower Mekong River. Contrary to common expectation, however, our long-term nutrient data show that bioavailable nutrient concentrations downstream have not declined after the building of the dams (Fig. 1). It is true that increasing trends for both DIN and bioavailable P might be partially caused by elevated anthropogenic nutrient inputs in the drainage basins of the reservoirs during the investigated period, e.g. by intensified agriculture and an expanding human population. However, our study clearly indicates that the bioavailability of nutrients increased after passing through the cascade reservoirs, as shown by the gradually increasing DIN concentration as well as the ammonium/DIN and bioavailable P/total P ratios from dam to dam.

**Figure 5.**
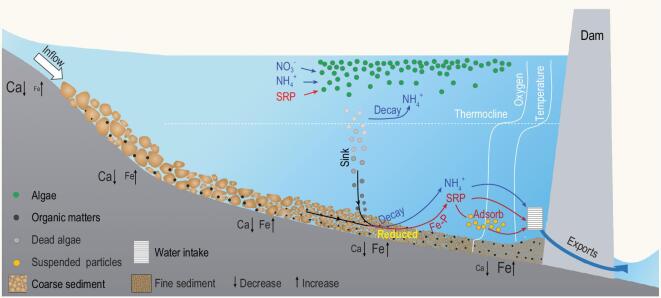
The conceptual mechanism for the stimulation of phytoplankton production and modification of nutrient export downstream by dams in the upper Mekong River.

The construction of hydropower reservoirs triggers a series of physical and chemical changes that lead to an increase in nutrient bioavailability downstream. These changes included increasing phytoplankton densities caused by longer hydraulic residence time, an increase in sedimentation of autochthonous organic matter, development of hypoxic water above the sediment, release of soluble reactive P from the sediment, transformation from nitrate to ammonium, release of ammonium and finally increased downstream export of these bioavailable nutrients in water discharged from the bottom of the dam (Fig. 5). Previous studies reported that many newly constructed reservoirs experienced upsurges in nutrients (‘trophic surge’) released from the inundation of terrestrial soils [33]. However, in the catchment of the upper Mekong basin, there was little soil but rocks with limited nutrient release after the impoundment. We therefore attribute the increase of bioavailable nutrients in reservoirs to the alteration of biogeochemical nutrient cycles due to the hydropower dams.

This work implies that the fishery collapse in the lower Mekong River might be caused by other factors such as overfishing, habitat modification, disruption of fish migration or water-quality deterioration from local sources [8,34], rather than a reduction in nutrient availability or reduced primary productivity induced by the cascade dams upstream. Although the construction of reservoirs can have major negative ecological consequences for a range of processes such as greenhouse-gas emissions or fish migration [35,36], they do not inevitably lead to a reduction in the concentration and form of dissolved nutrients downstream. On the contrary, excessive riverine exports of bioavailable nutrients might cause potential negative consequences of increased productivity in stagnant waters downstream and coastal waters, leading to risks of eutrophication and oxygen depletion.

Our results illustrate the value of dedicated monitoring programs, which may reveal changes in nutrient dynamics, primary productivity and species composition that were not foreseen by modeling scenarios. Although our study investigated and compared multiple hydropower dams, it was limited to the upper Mekong. Extending these monitoring efforts to the lower Mekong may result in an even more integrative understanding of the environmental impacts of dams and reservoirs in large river systems. An improved scientific understanding of these environmental impacts is much needed, given that hydropower exploitation can contribute to energy supply and poverty alleviation in economically less developed regions such as the lower Mekong and Congo basin.

## CONCLUSION

It is commonly assumed that hydropower dams retain nutrients and therefore reduce downstream primary productivity. Our long-term data and extensive studies on nutrient cycling as well as phytoplankton-community dynamics in the cascade reservoirs along the upper Mekong River demonstrated that hydropower reservoirs increased downstream bioavailability of nitrogen and phosphorus, stimulated phytoplankton abundance and shifted the dominant species from diatoms in the silicon-rich upstream channel to green algae in downstream reservoirs. The core mechanism is the synergic effect of increased hydraulic residence time and the development of hypoxic conditions due to stratification and organic-matter accumulation causing an increase in the concentration of nutrients at depth that are released downstream from the base of the reservoir. Our findings produce a new perspective on the environmental impacts of hydropower reservoirs on nutrient cycling and ecosystem functioning, with potential implications for sustainable development of hydropower worldwide.

## MATERIALS AND METHODS

### Study area

**Figure 6.**
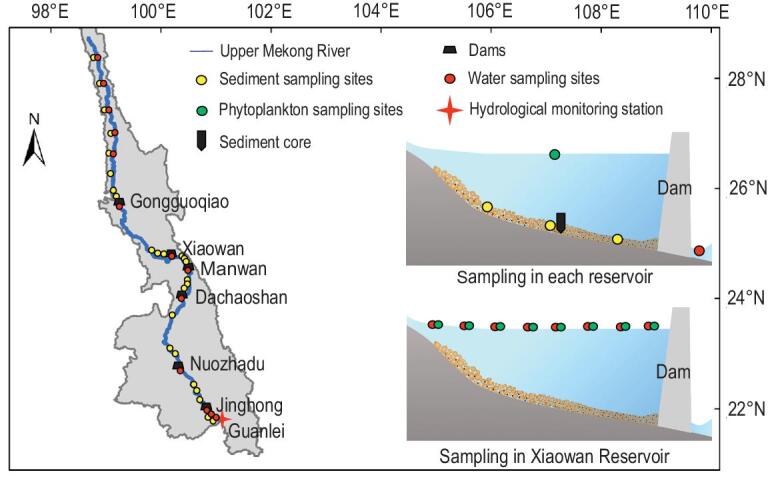
Location of cascade dams and sampling sites in this study. The gray shading shows the catchment of the upper Mekong River.

The Mekong River, with a length of 4909 km, is the twelfth longest river in the world and the seventh longest in Asia. It drains an area of 760 000  km^2^, discharging 457 km^3^ of water annually to the ocean [37]. The Mekong basin can be divided into two parts: the ‘upper Mekong basin’ in China and the ‘lower Mekong basin’ from Yunnan in China to Southeast Asia. The most precipitous drop in the Mekong River occurs in the upper basin, where it falls 4500 m to create rich hydropower resources. The river has a small rocky catchment with little soil [38]. By 2016, six dams had been built for hydropower production in the upper Mekong River in China, including Gongguoqiao, Xiaowan, Manwan, Dachaoshan, Nuozhadu and Jinghong. The locations of these dams are shown in Fig. 6. Hydraulic residence time (yr) was calculated as *V*/*Q*, where *V* is the reservoir volume (m^3^) and *Q* is the mean annual flow rate from the dam (m^3^ yr^−1^). The main features of the cascade reservoirs are presented in Table 1 in the Supplementary data.

### Field survey

Two field surveys were conducted in the upper Mekong River in China, stretching a length of 1200 km, in September 2016 and September 2017. The reasons to conduct the field surveys in late summer were the following: (i) the study area has no distinct seasonality with respect to hydrologic regime; (ii) in aquatic ecosystems, dynamics of major nutrients (N and P) and phytoplankton are closely coupled; and (iii) phytoplankton prefers to proliferate in warm period. In the field survey, there were five sampling sites in the upstream channel of the first cascade and two sampling sites in the downstream channel of the last cascade. In the upstream and downstream channels, the distance between two neighboring sites was about 50 km. There were three sampling sites in each reservoir, considering the spatial heterogeneity of hydrodynamic and thereby sedimentation conditions, and also one site near the outlet of the power station right after each dam (Fig. 6). Water and suspended particles were collected from the sampling sites in the upstream and downstream channels, as well as the sampling sites near the outlet of the power station right after a dam. Sediment and phytoplankton were collected at the sampling sites in the upstream and downstream channels, as well as in reservoirs. Intact sediment cores were collected at the sampling sites in reservoirs. To further investigate the transformation mechanism between nutrient and phytoplankton within a reservoir, nutrients in water were dedicatedly obtained from eight sampling sites in the Xiaowan Reservoir.

Field surveys were conducted using a synchronous-sampling strategy by several workgroups (see Fig. 7 in the Supplementary data). There were two workgroups for the five sites in the upstream channel, one workgroup for three sites in each reservoir and one site right after the dam, and one workgroup for the two sites in the downstream channel. Both the dedicated water samples in Xiaowan Reservoir and the sediment cores were collected by a specific work group.

At each site, water and sediment were collected randomly in triplicate using a stainless-steel bucket and an Ekman grab sampler, respectively. They were homogenized completely, and then part of the homogenized samples was taken randomly as the sample of this site. A water sample of 500 mL was frozen in acid-washed PVC bottles for nutrient analysis and a water sample of 1000 mL was preserved with 1% Lugol's solution and concentrated to 30 mL after 48-h sedimentation for phytoplankton-community analysis. Sediment samples were frozen for the analyses of organic carbon and bioavailable P. Suspended particles were collected using a wide-mouth bottle to analyse particulate bioavailable P. The wide-mouth bottle was deployed vertically 10 m below the water surface for 1 month from 1 September to 30 September in each survey to collect suspended particles by natural settling. Intact sediment cores were collected using an automatic deep-water sampler (see Fig. 6 in the Supplementary data) to analyse nutrient fluxes across the sediment–water interface according to Zhang *et al.* [39].

The existing data sets of nutrients (N and P) during 1983–2018 at the trans-boundary monitoring station (Fig. 1) were from Chinese Hydrological Year Book and the Hydrology and Water Resource Bureau of Yunnan Province, China. The data sets have three times (high flow, normal flow, low flow) of records per year in the 1980s, four values (somehow seasonally) in the 1990s and monthly values since 2000. The existing data sets of installed capability and hydropower production of the cascade reservoirs were from China Huaneng Group Co., Ltd.

### Sample analysis

Concentrations of suspended particles, nitrate, ammonium, nitrite and soluble reactive P in the water column were analysed according to the Monitoring Analysis Method of Water and Wastewater [40]. Dissolved silicon was analysed by a Bran-Luebbe AAIII Autoanalyzer using the silicomolybdic blue method [41]. Total P in bed sediments and suspended particles were analysed after acid hydrolysis at high temperature (340°C) according to Murphy and Riley [42]. The P fractions were determined on wet samples using the sequential-extraction method [43]. The sequential extraction was as follows: 1 M ammonium chloride (NH_4_Cl) at pH 7.0, 0.11 M sodium hyposulfite (Na_2_S_2_O_4_)/0.11 M sodium bicarbonate (NaHCO_3_), 0.1 M sodium hydroxide (NaOH) with a further digestion, 0.5 M hydrogen chloride (HCl), the measured P fraction of which was referred to as exchangeable P, iron-bound P, aluminum-bound P, organic-bound P and calcium-bound P, respectively. Bioavailable P refers to exchangeable P, iron-bound P, aluminum-bound P and organic-bound P, and the remaining fraction is referred to as Non-bioavailable P, including calcium-bound P and residual P determined from the difference between total P and the sum of the different fractions. Sediment organic carbon was analysed using a vario MACRO cube elemental analyser (Elementar Inc., Germany) after the fresh sediment was freeze-dried and ground. Phytoplankton were quantified using a light microscope (Olympus BX41) at 400× magnification. The units (cells, colonies and filaments) were enumerated in random fields and ≤200 individuals of the most frequent species were counted [44]. Phytoplankton species were identified according to Hu and Wei [44] and John *et al.* [45].

### Statistical analysis

Mean values for sites were calculated (geometric mean in the case of pH). One-way ANOVA was employed to test the statistical significance of differences between sampling sites. Post-hoc multiple comparisons of treatment means were performed using the Tukey's least significant difference procedure. All statistical analyses were carried out using SPSS v22.0. The level of significance was *P* < 0.05 for all tests.

## Supplementary Material

nwaa026_Supplemental_File
