# Supplementary material for: Hydropower reservoirs on the upper Mekong River modify nutrient bioavailability downstream
Source: Natl Sci Rev. 2020 Feb 17;7(9):1449–57. doi: 10.1093/nsr/nwaa026 (PMC8288771; doi:10.1093/nsr/nwaa026)
Supplement: nwaa026_Supplemental_File [file nwaa026_supplemental_file.docx]

**Supplementary data**

**Hydropower reservoirs on the upper Mekong River modify nutrient bioavailability downstream**

Qiuwen Chen^1,2,†,^*, Wenqing Shi^1,2,†^, Jef Huisman^3^, Stephen C. Maberly^4^, Jianyun Zhang^5^, Juhua Yu^2^, Yuchen Chen^2^, Daniele Tonina^6^, Qitao Yi^2^

^1^State Key Laboratory of Hydrology-Water Resources & Hydraulic Engineering, Nanjing Hydraulic Research Institute, Nanjing 210029, China.

^2^ Center for Eco-Environment Research, Nanjing Hydraulic Research Institute, Nanjing 210098, China.

^3^Department of Freshwater and Marine Ecology, Institute for Biodiversity and Ecosystem Dynamics, University of Amsterdam, Amsterdam, The Netherlands.

^4^Lake Ecosystems Group, UK Centre for Ecology & Hydrology, Lancaster LA1 4AP UK.

^5^Yangtze Institute for Conservation and Development, Nanjing 210029, China.

^6^Center for Ecohydraulics Research, University of Idaho, Boise, ID 83702, USA.

^*^Corresponding author: Tel./Fax: +86 2585829765; E-mail: [qwchen@nhri.cn](mailto:qwchen@nhri.cn).

^†^These authors contributed equally to the paper.


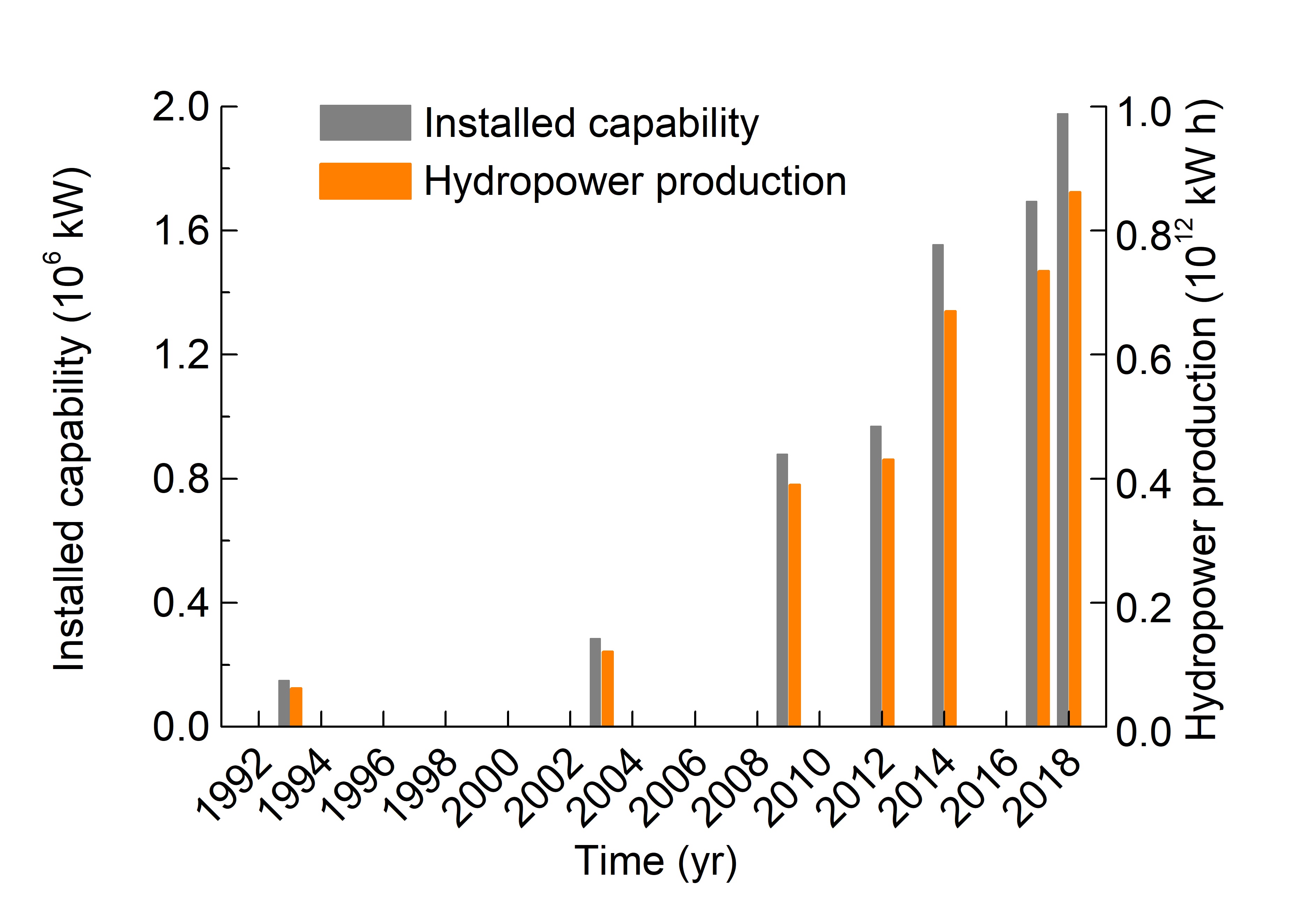


**Figure 1** The development of hydropower in the upper Mekong River. The data were collected from China Huaneng Group Co., Ltd.


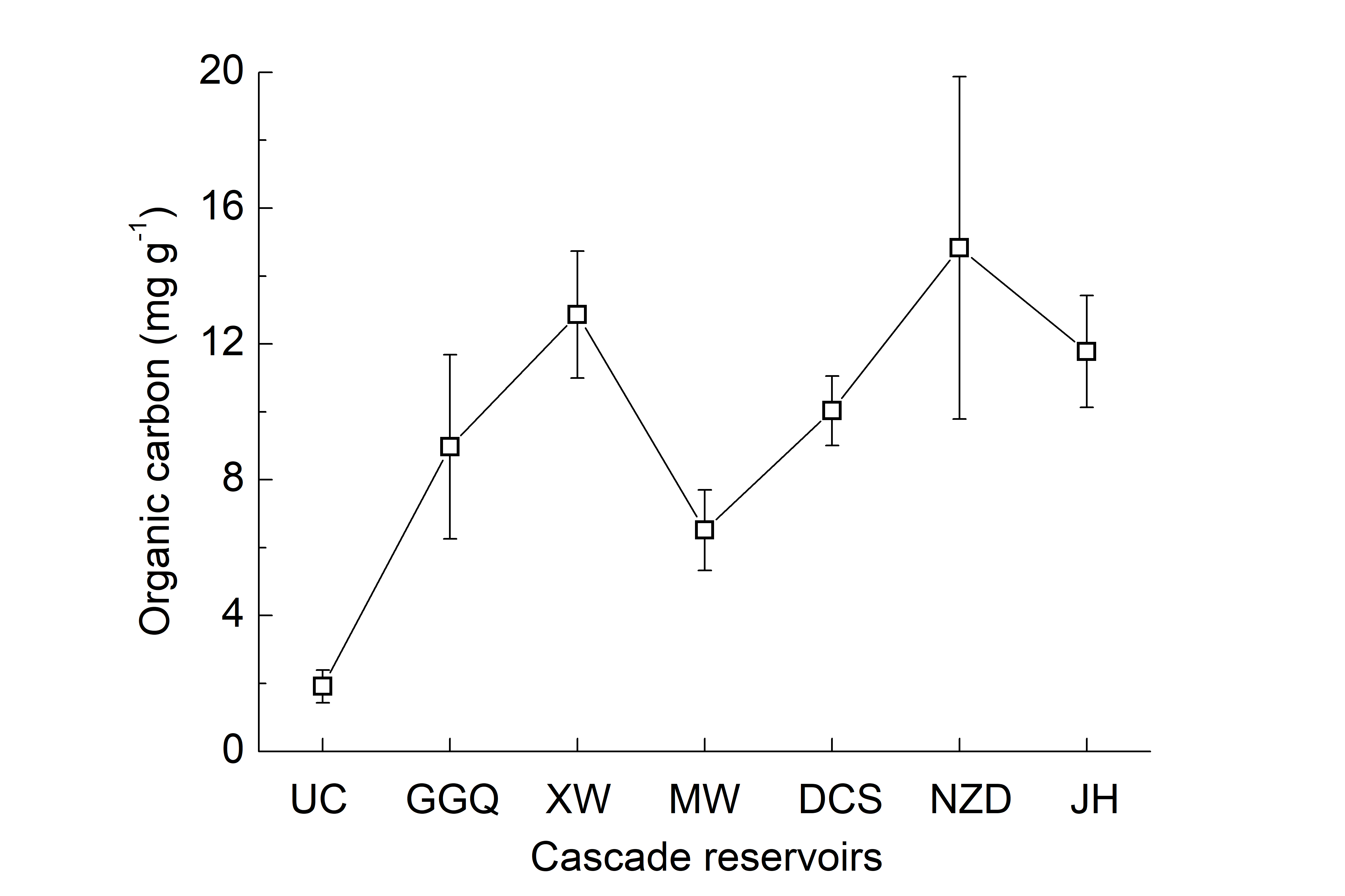


**Figure 2** Organic carbon content in bed sediments along the upper Mekong River. GGQ (Gongguoqiao), XW (Xiaowan), MW (Manwan), DCS (Dachaoshan), NZD (Nuozhadu) and JH (Jinghong) are cascade reservoirs along the flow direction in the upper Mekong River. UC = upstream channel. Data were collected in September 2016 and 2017. Error bars indicate standard deviations (n = 3).


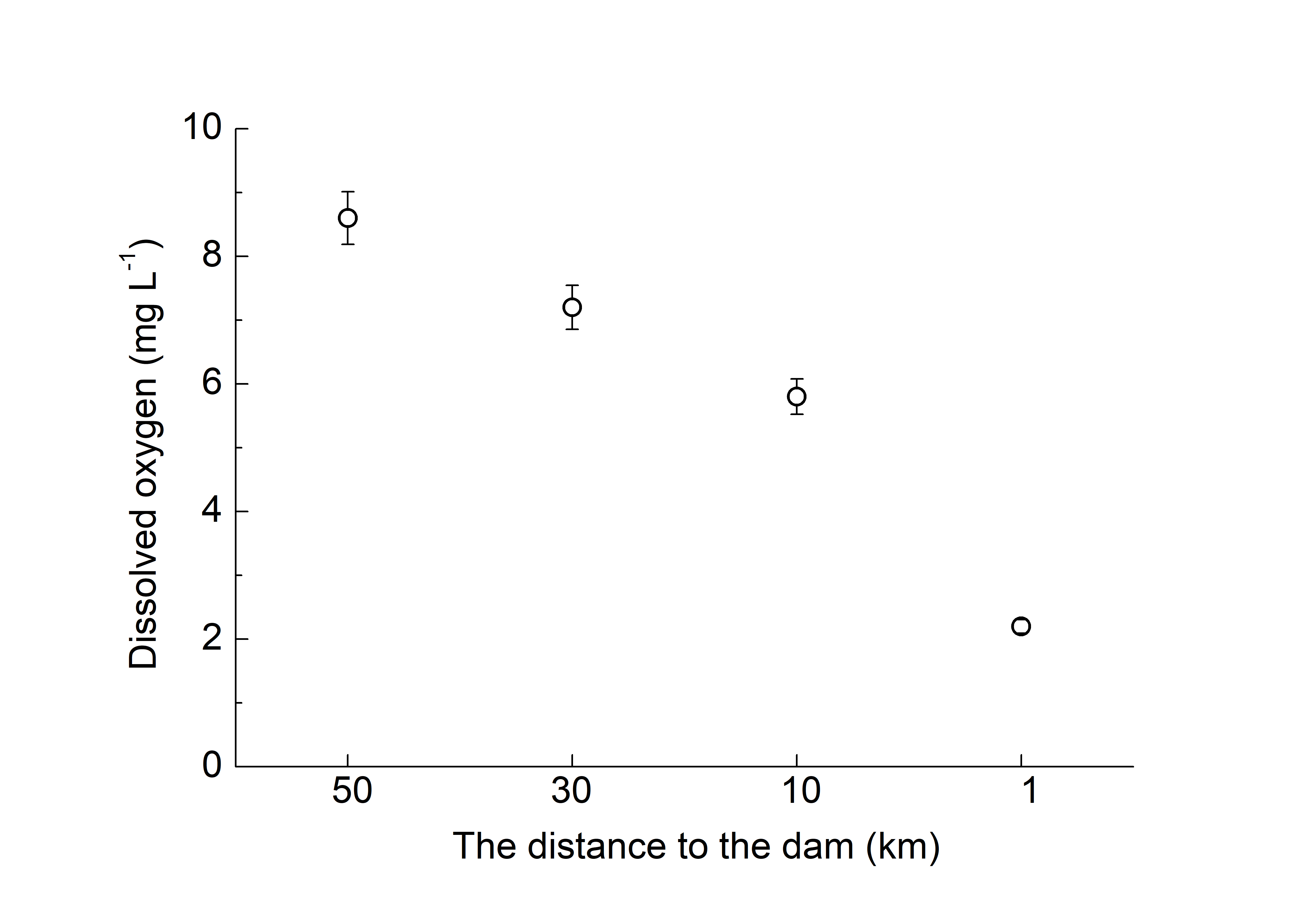


**Figure 3** Dissolved oxygen concentration in the hypolimnion of the Gongguoqiao Reservoir in the upper Mekong River. Data were collected in September 2016 and 2017. Error bars indicate standard deviations (n = 3).


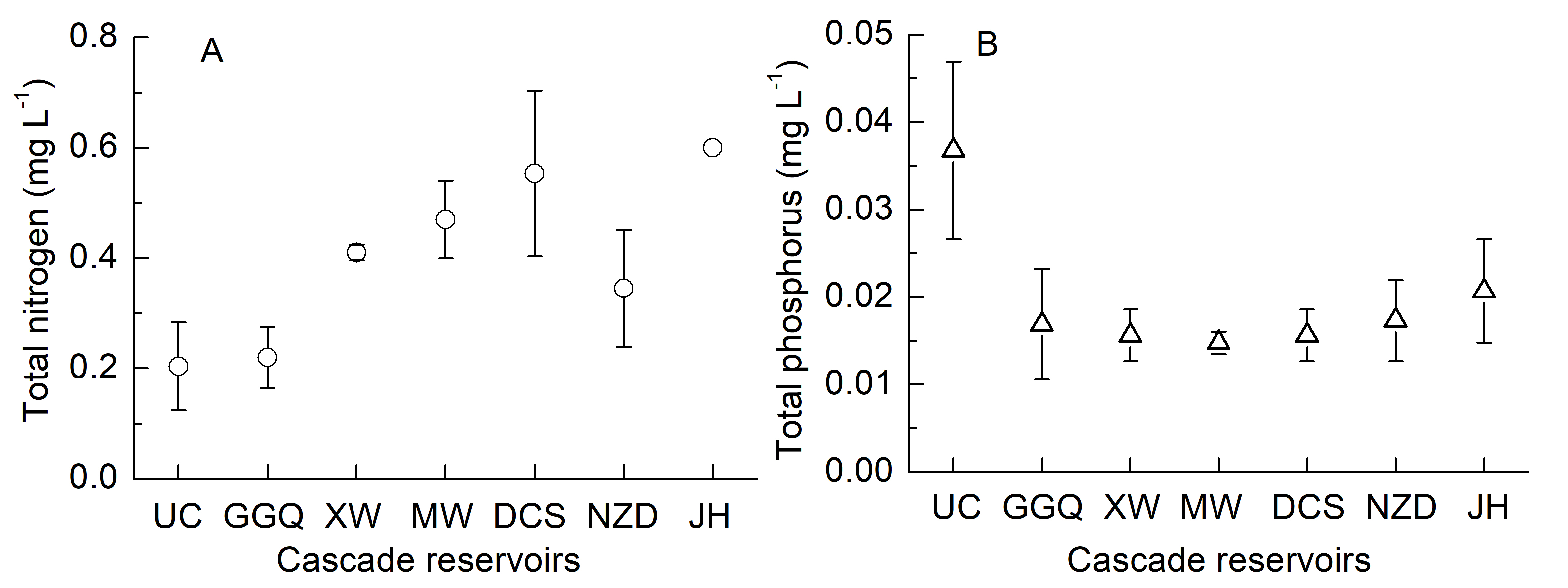


**Figure 4** Total nitrogen and total phosphorus in the surface water in cascade reservoirs along the upper Mekong River. (A) Total nitrogen; (B) Total phosphorus. GGQ (Gongguoqiao), XW (Xiaowan), MW (Manwan), DCS (Dachaoshan), NZD (Nuozhadu) and JH (Jinghong) are cascade reservoirs along the flow direction in the upper Mekong River. UC = upstream channel. Data were collected in September 2016 and 2017. Error bars indicate standard deviations (n = 3).


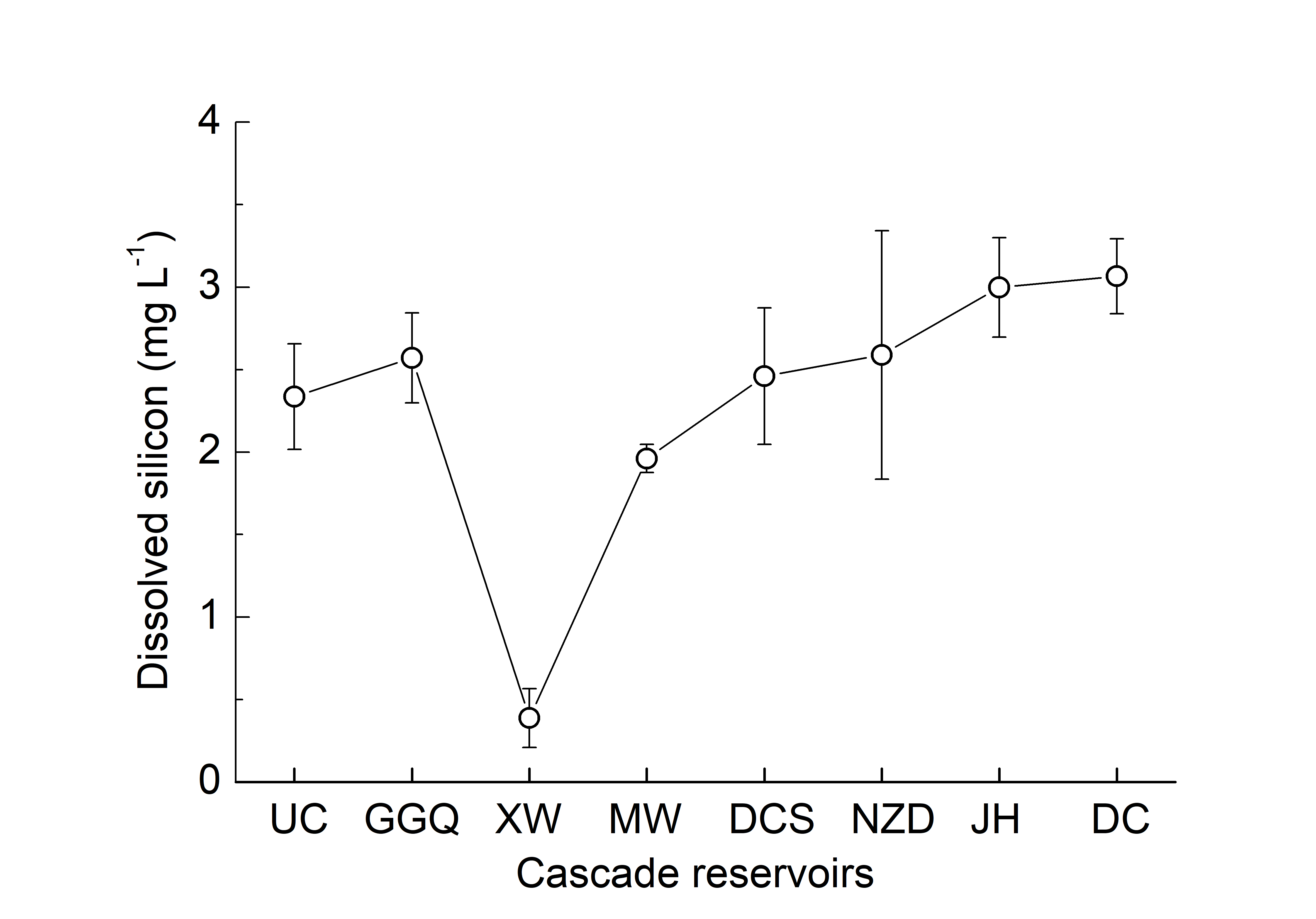


**Figure 5** Dissolved silicon concentration in the surface water in cascade reservoirs along the upper Mekong River. GGQ (Gongguoqiao), XW (Xiaowan), MW (Manwan), DCS (Dachaoshan), NZD (Nuozhadu) and JH (Jinghong) are cascade reservoirs along the flow direction in the upper Mekong River. UC = upstream channel, DC = downstream channel. Data were collected in September 2016 and 2017. Error bars indicate standard deviations (n = 3).

**
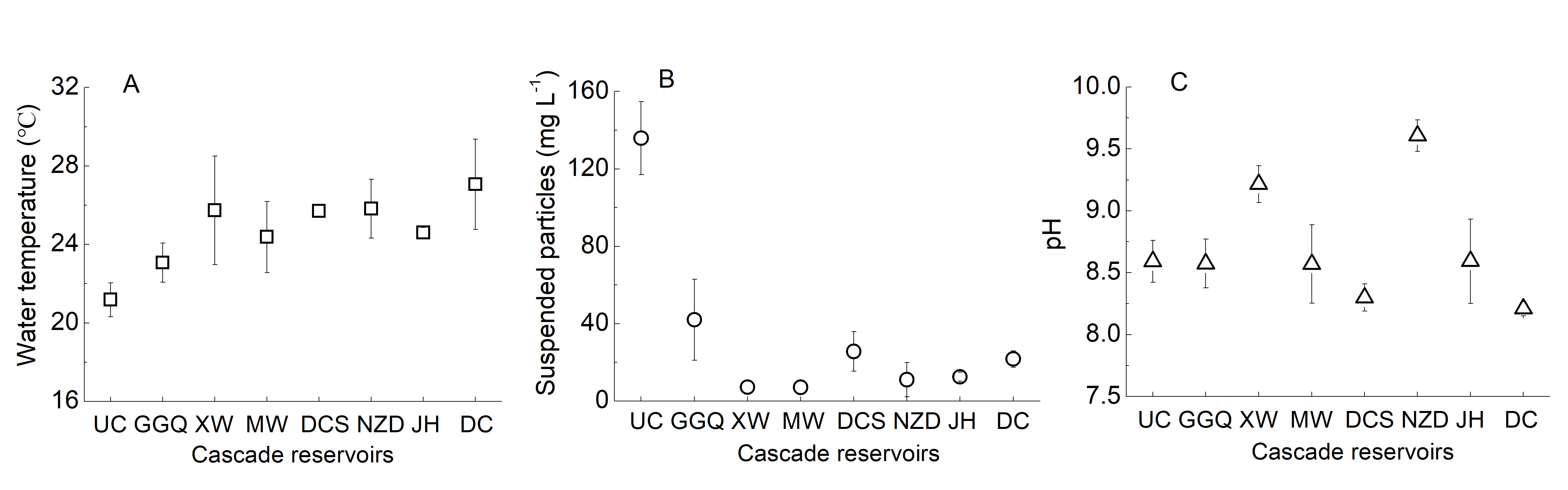
**

**Figure 6** Changes in physicochemical properties of the water in cascade reservoirs along the upper Mekong River. (A) Water temperature; (B) Suspended particles; (C) pH. GGQ (Gongguoqiao), XW (Xiaowan), MW (Manwan), DCS (Dachaoshan), NZD (Nuozhadu) and JH (Jinghong) are the cascade reservoirs along the flow direction in the upper Mekong River. UC = upstream channel, DC = downstream channel. Data were collected in September 2016 and 2017. Error bars indicate standard deviations (n=3).


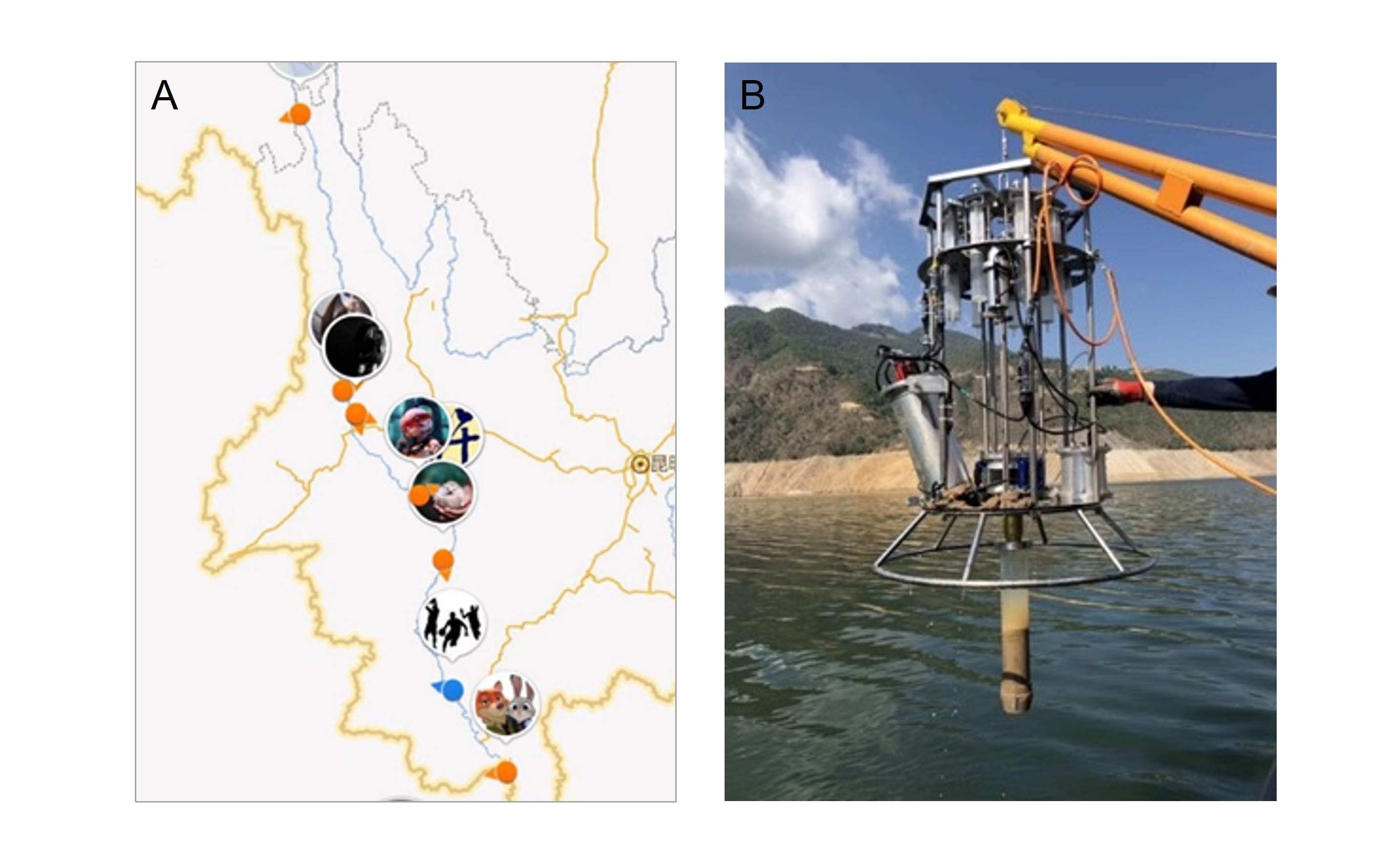


**Figure 7** Sample collections in the field surveys. (A) The illustration of simultaneous sampling of water and sediments at different sites along the river. The figure is a screen capture of position sharing in Baidu Map during simultaneous sampling. The small cartoons along the river in the map are the mobile phone IDs of the sample collectors for simultaneous sampling. (B) Sampling of intact sediment cores in the reservoirs with a maximum water depth of over 280 m.

**Table 1** The main features of cascade hydropower reservoirs in this study

|  | GGQ | XW | MW | DCS | NZD | JH |
| --- | --- | --- | --- | --- | --- | --- |
| Dam height (m) | 105 | 292 | 132 | 115 | 261.5 | 108 |
| Water level (m) | 1307 | 1240 | 994 | 899 | 812 | 602 |
| Storage capacity (10^8^ m^3^) | 3.5 | 149.1 | 5.0 | 9.4 | 237.0 | 11.4 |
| Discharge volume (10^8^ m^3^) | 318.5 | 381.6 | 388.0 | 419.0 | 545.6 | 574.0 |
| Installed capacity (10^6^ kW) | 0.90 | 4.20 | 1.50 | 1.35 | 5.85 | 1.75 |
| Hydraulic residence time (yr) | 0.01 | 2.36 | 0.78 | 0.30 | 1.87 | 0.40 |
